# *Ab-initio* and Conformational Analysis of a Potent VEGFR-2 Inhibitor: A Case Study on Motesanib 

**Published:** 2014

**Authors:** Ahmad Ebadi, Nima Razzaghi-Asl, Sara Shahabipour, Ramin Miri

**Affiliations:** a *Medicinal and Natural Products Chemistry Research Center, Shiraz University of Medical Sciences, Shiraz, Iran.*; b *Department of Medicinal Chemistry, School of Pharmacy, Shiraz University of Medical Sciences, Shiraz, Iran.*; c *Department of Medicinal Chemistry, Schoolof Pharmacy, Ardabil University of Medical Sciences. *

**Keywords:** VEGFR-2, Motesanib, Cancer, B3LYP, DFT

## Abstract

Vascular endothelial growth factor receptor-2 (VEGFR-2); a cell surface receptor for vascular endothelial growth factors, is a key pharmacological target involved in the cell proliferation/angiogenesis. It has been revealed that VEGFR-2 induces proliferation through activation of the extracellular signal-regulated kinases pathway. In this regard, targeting the VEGFR-2 has been considered as an efficient route to develop anti-tumor agents. Motesanib is a small-molecule antagonist of VEGFR-1, 2, and 3 (IC50s; 2 nM, 3 nM, 6 nM, respectively). It is an experimental drug candidate undergoing clinical trials against some types of cancer. In the present study, Motesanib (AMG 706) was evaluated in terms of its binding energies with individual amino acids of VEGFR-2 active site (amino acid decomposition analysis). For this purpose, functional B3LYP associated with split valence basis set using polarization functions (Def2-SVP) was used. Comparative conformational analysis of the ligand in optimized and crystallographic states revealed that Motesanib does not necessarily bind to the VEGFR-2 active site in its minimum energy conformer.

## Introduction

Vascular endothelial growth factors (VEGFs) bind to three structurally related receptor tyrosine kinases; VEGFR-1, VEGFR-2, and VEGFR-3. A number of coreceptors such as neuropilins that lack intrinsic catalytic activity bind to VEGF and also modulate the effect of the VEGFRs (1). VEGFRs have a high degree of homology within the kinase domain; however, their signaling properties greatly differ (1). VEGFR-2 is the major mediator of responses in endothelial cells and it is considered to be a principal signal transducer in angiogenesis (2). Vascular endothelial growth factor receptor 2 (VEGFR-2) is a cell surface receptor for VEGFs (3,4). VEGF signaling pathway has been well demonstrated to induce angiogenesis during tumor development (5,3). Vascular endothelial growth factor (VEGF) has been found to be a major driver of tumor angiogenesis leading to efforts in development of novel therapeutics aimed at inhibiting its activity. It is generally accepted that the destructive growth of tumors and their metastases is highly depended on angiogenesis (6).

Anti-VEGF therapy has been regarded as a prominent choice for the management of several human malignancies. The vast majority of solid tumors are involved with VEGF overexpression (7). In this regard, chemical agents with the inhibitory activity on angiogenesis may be focused as a workable treatment options for patients involved with solid tumors (8). Inhibition of VEGF has been reported to significantly suppress tumor angiogenesis in mouse tumor models (9). The angiogenesis process of solid tumors may be avoided due to the inhibition of the tyrosine kinase VEGFR-2 signaling pathway. In this way required blood flow for developing tumor would decrease dramatically and even the growth will stopped because of lack of nutrient and growth factors supported by freshly forming vessels (10,11). Hence, anti-tumor drug development targeting VEGFR-2 signalling pathway has been recently highlighted as an important way in the clinical trials (12). 

A number of structurally diverse small molecule VEGFR-2 inhibitors such as indolin-2-ones, phthalazines, quinolinones, imidazopyridines, benzimidazoles,quinoline amides, pyridines and quinazolines have been introduced in the literature (13-19). In a typical lead/drug discovery protocol, potential candidate molecules may suffer from undesirable properties in their pharmacokinetic and pharmacodynamic profiles. Structure-based drug design has been considered as one of the major strategies in achieving potential drug candidates (20,21). Motesanib (N-(3,3-dimethyl-2,3-dihydro-1H-indol-6-yl)-2-((pyridin-4-ylmethyl)amino)pyridine-3-carboxamide) is an experimental drug candidate which has been exhibited antagonistic activity for VEGFR-2 (22). Clinical trials on potential beneficial effects of Motesanib against some types of cancers are being actively pursued. Results showed that Motesanib inhibited VEGFR-1, 2, and 3 with IC50 values of 2, 3 and 6 nM respectively (23-25). 

In lead-drug development strategies, combination of experimental methods with computer aided molecular design (CAMD) techniques is essential for the development of new drugs aimed at new targets, and thus for medicinal chemistry (26). Various computational chemistry methods are in hand for running CAMD. These methods comprise mainly two categories; ligand- based and structure-based methods. Within this scenario, theoretical methods based on density- functional theory (DFT) are going to play an increasingly important role in many applications of computational chemistry to drug discovery (27-29). One of the mostly used DFT methods is Becke three-parameter Lee-Yang-Parr (B3LYP) hybrid density functional theory (30). This technique has been applied for conformational analysis of antiangiogenic agents such as Motesanib (31). Estimating the proportion of individual amino acid-ligand interaction energies in total binding energy would be a very useful trend in pharmacophore discernment and development (Amino acid decomposition analysis). No such report could be found for Motesanib with VEGFR-2. 

In continuation to our interest in developing new cytotoxic agents (32), we aimed to determine the binding pose and binding energies of Motesanib in VEGFR-2 active site. For this purpose, DFT calculations at the level of B3LYP/Def2-SVP were used to estimate the individual amino acid-ligand interaction energies. Furthermore; conformational analysis of Motesanib was performed at the same level of theory to determine the torsional deviation from minimum energy state upon binding to the receptor.

## Experimental

*Computational section*

X-ray crystallographic structure of VEGFR-2 with its cognate ligand (AMG 706) was downloaded from Brook Haven Protein databank (PDB code: 3EFL, www.rcsb.org .(All the pre-processing procedure for ligand and receptor crystallographic files were done within WHAT IF server (European Molecular Laboratory Heidelberg, Germany). All hydrogens were properly added to the receptor PDB file using What if server. All computational DFT studies were performed using functional B3LYP associated with split valence basis set using polarization functions (Def2-SVP) (33).

The evaluated amino acid residues in *ab initio *study (Leu889, Ala866, Lys868, Glu885, Val899, Val916, Cys919, Leu1035, Asp1046) were chosen according to the information from crystallographic data (34). The proposed interaction profile could be found in Brook Haven Protein databank. All amino acids were considered in their real electrostatic state. For each residue under study, N-terminal was acetylated and C-terminal was methyl amidated to mimic the original electron density. All conformational and configurational features were the same as the X-ray structure. However the positions of hydrogen bonds are not clearly recognized in a typical X-ray crystallographic file and this restriction persuaded us to further optimize the heavy atom-hydrogen bonds using constrained optimization method (heavy atom fixing approximation).

All ligand-receptor interaction energies were estimated by B3LYP/ Def2-SVP method. The whole calculations were done with the ORCA quantum chemistry package (30). The optimized structures were visualized by Visual Molecular Dynamics program (VMD) (35). 

## Results and Discussion

*Ab initio study of ligand-receptor interactions*

**Figure 1:**
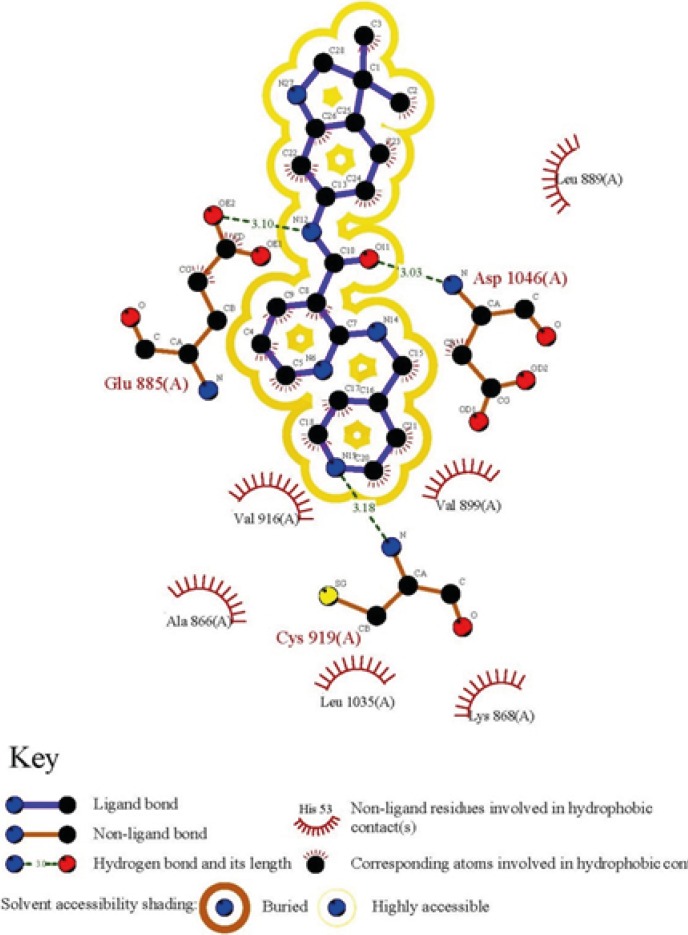
2D representation of ligand-receptor interactions for Motesanib in VEGFR-2 active site

To obtain a binding profile between a Motesanib and VEGFR-2 active site, relevant amino acids were chosen on the basis of information from Protein Data Bank (PDB). The representation of the Motesanib structure in the active site of VEGFR-2 was further confirmed via schematic 2D interaction profile generated by LIGPLOT Figure 1.

The directionality of hydrogen bonds including optimum distances and angles supports efficient interactions with receptor. Bearing this in mind, the optimization process was done with the same basis set to obtain the exact geometry of H-bonds. The related data are summarized in Table 1. Hydrogen bond geometries were described as H-donor-acceptor angles. It should be noted that hydrogen bond lengths were obtained considering H-acceptor distances. 

**Table 1:** Hydrogen-bond analysis of cognate inhibitor (Motesanib) with VEGFR-2 residues

**Amino acid**	**Optimized hydrogen bond distances (Å) state**	**hydrogen bond angle (degree)**
Glu885	2.11	11.66
Asp1046	2.04	9.60
Cys919	2.19	5.21

Ligand-residue binding energies (ΔE_b_) were calculated using the following equation: 

Equation (1) $${∆E}_{b}=E_{\mathrm{LR}}-E_{L}-E_{R}$$

**Figure 2:**
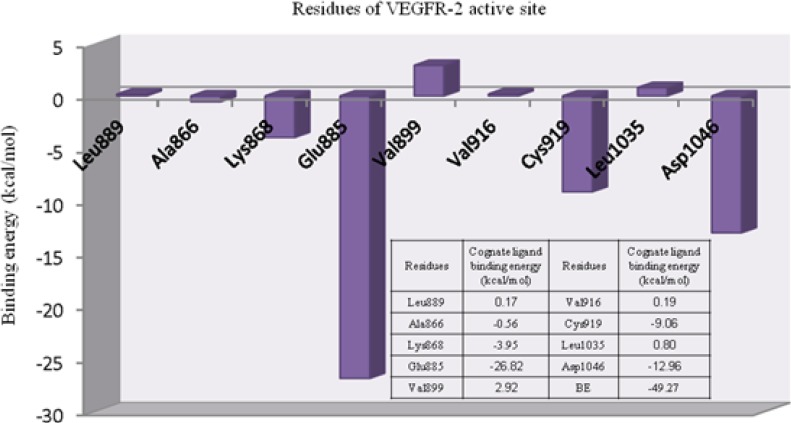
Individual residue-ligand interaction energies for Motesanib and VEGFR-2.

E_LR_ stands for residue-ligand interaction energy, E_R_ and E_L_ indicate the electronic energies for residues and ligand, respectively. Individual ligand-residue binding energies are shown in Figure 2. 

Lipophilic contacts are a function of orientation, constant/induced dipole moments and distance (36). Attractive hydrophobic interaction was made between AMG 706 and Ala866 residue (-0.56 cal/mol). Regarding the obtained results, one may assume that electrostatic hydrogen bonding interactions may have significant contribution to total binding energy between AMG 709 and receptor. For Leu889, Val899, Val916 and Leu1035 the positive binding energies might be related to an inappropriate orientation of ligand in the active site of receptor (crystallographic state). It should be noticed that molecular dynamic may assist in balancing these close contacts which are responsible for repulsive interactions with receptor.

**Figure 3:**
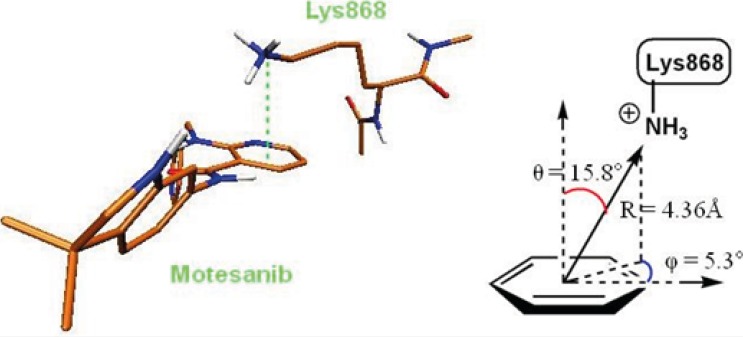
3D representation of cation-π interaction between Lys868 and Motesanib

The role of cation-pi interaction as a force for molecular recognition in biological media has been revealed via studies on model systems and the analysis of biological macromolecular structures (36). In Lys868 the quaternary amine moiety might be responsible for observed cation-π interaction with central pyridine ring of AMG 709 (-3.93 kcal/mol). Binding pose of the ligand revealed that nitrogenous cation centered on the top of the π face of pyridine ring (Figure 3). Our estimated energy for the associated cation-π interaction correlated well with previously reported data (37), but might be less than accepted values (38). This energy difference might be attributed to the existence of pyridine lone pair. In pyridine, the lone pair does not participate in aromaticity and thus electronegativity of the heteroatom wins out and weakens the cation-π binding ability. 

The deprotonated Glu885 is the most significant residue for enzyme-inhibitor interactions due to the strong hydrogen bond with the amide NH in AMG 706. Typical charge-assisted hydrogen bond between Glu885 and amide NH was found to be supported by significant binding energy of -26.82 kcal/mol. Another important H-bond between Asp1046 and amide oxygen was associated with -12.96 kcal/mol interaction energy. In the case of Cys919 residue, the observed binding energy was estimated to be -9.06 kcal/mol. In fact hydrogen bonding interaction with Cys919 from H-donor group of the inhibitor is the key feature of VEGFR inhibitors (39).

In ligand-receptor interaction, stereoelectronic effects are prominent in determining complementary potential electrostatic surfaces. Ligand electronic structure may address its proper orientation in the enzyme active site and potent inhibition would be expected regarding proper fitness of the ligand and electronic surfaces of the active site. 

**Figure 4:**
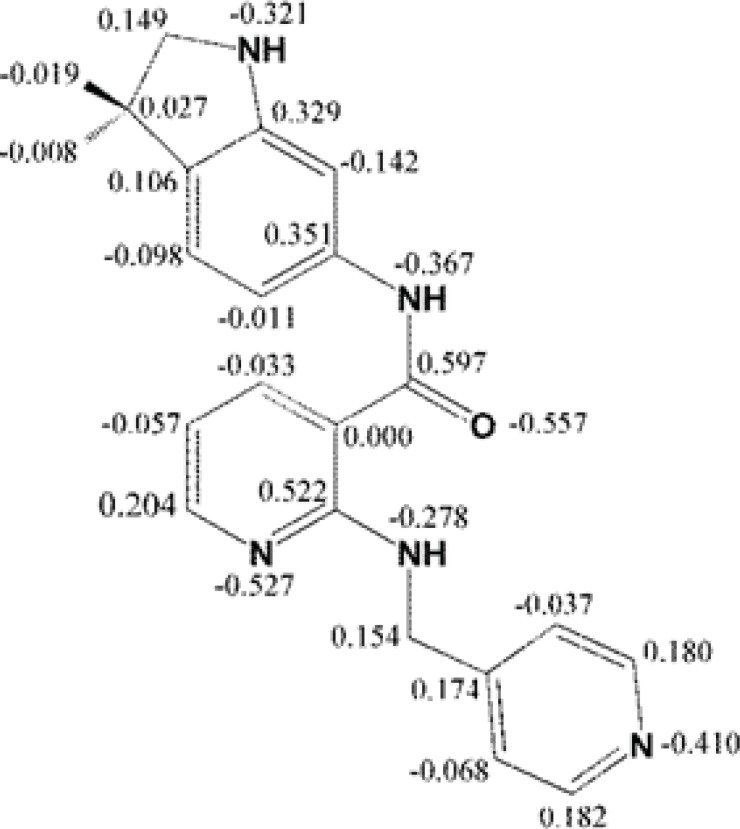
Mulliken partial charge distribution for Motesanib heavy atoms

Mulliken partial electronic charges were assigned to the constituent atoms of compound AMG 709 (Figure 4) (40). It should be noted that atoms participated in key bindings with Asp1046, Glu885 (charge-assisted interactions) and Cyc919 residues possessed relatively negative electronic charges. 

*Comparative conformational analysis*

We decided to quantify the conformational divergence of AMG 709 upon binding to the VEGFR-2 active site. For this purpose, aqueous biological medium was modeled in our ligand optimization procedure. Estimated binding energies for compound AMG 709 may be a direct outcome of varied internal energies of ligand in its protein bound and free states within biological media (ΔE_instability_). ΔE_inst._ can be defined as an energy difference for ligand in its free and protein bound states within aqueous medium. ΔE_inst._ needs to be considered in order to adjust obtained binding energies. Water was selected as a biological medium for this purpose. 

For the purpose of calculating ΔE_inst._, optimum structural conformation of compound AMG 709 was obtained in water and relevant energy was assigned to the free state . In the next step, the energy of receptor bound ligand was obtained in the crystallographic state. ΔE_inst._ may be well related with the free energy of binding via following equations:

Equation (2) $$∆G_{b}=∆H_{b}-T∆S_{b}$$

Equation (3) $$∆H_{b}=∆E_{t}-P∆V\approx ∆E_{\mathrm{tb}}$$

Equation (4) $$∆E_{\mathrm{tb}}=∆E_{b}+∆E_{\mathrm{inst.}}$$

**Figure 5:**
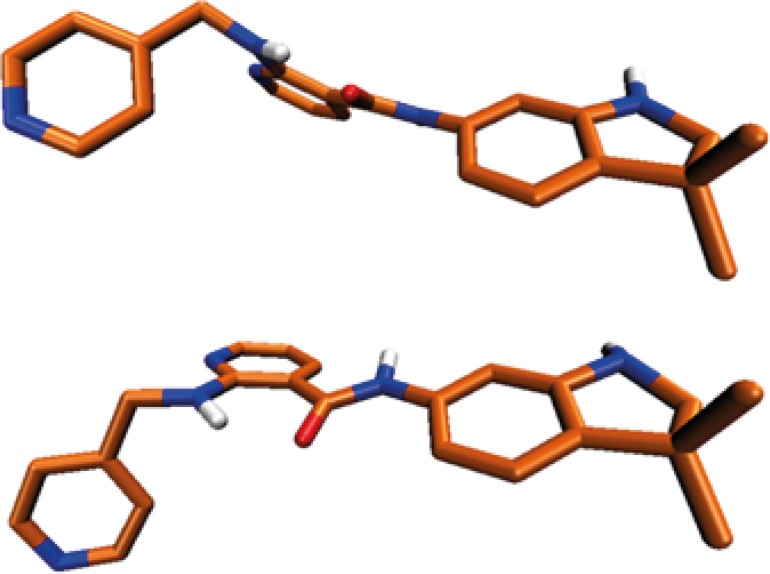
Conformational structure deviation of Motesanib in VEDFR-2 active site (up), and optimized conformer (down).

Higher ΔE_inst._ values support more positive total binding energies (ΔE_tb_) consequently leading to weaker ligand-receptor interactions in terms of free binding energies (ΔG_b_). Our calculations showed that AMG 709 tolerated 8.91 kcal/mol instability to gain the appropriate conformation in binding to the receptor. Based on the obtained results, ΔE_tb _was found to be -40.36 kcal/mol. Two conformational poses of the ligand are depicted in Figure 5.

However the difference between ΔE_tb _and ΔG_b_ values associated with relevant ligand may account for the participation of solvation in binding profile. In the light of the above information, solvation energy of Motesanib molecule needs must be taken into account for the correlation of ΔE_tb _and ΔG_b _terms. This result might further demonstrate the important role of solvent molecules in determining final free binding energy of ligand-receptor system. 

The estimated conformational change of ligand structure upon binding to the receptor was evaluated in a more detailed way via performing comparative conformational analysis of the molecular geometries. For this purpose, optimized 3D structure of AMG 709 was obtained by DFT calculations via B3LYP method in association with split valence basis set using polarization functions (Def2-SVP). Frequency calculation with same basis set was performed to confirm the optimized structure. All frequencies were real and no imaginary frequency was seen. The resulted geometric poses in terms of bond lengths and dihedral angles are summarized in Tables 2 and 3. It should be noticed that due to the uncertainty in the delicate position of hydrogen atoms in crystallographic file, associated data have not been shown in Tables. We found that all the calculated bond lengths of the DFT optimized structure were in adaptable correlation with the crystallographic data. 

**Table 2:** Bond lengths of Motesanib in the optimized and crystallographic (VEGFR-2, PDB code: 3EFL) conformers

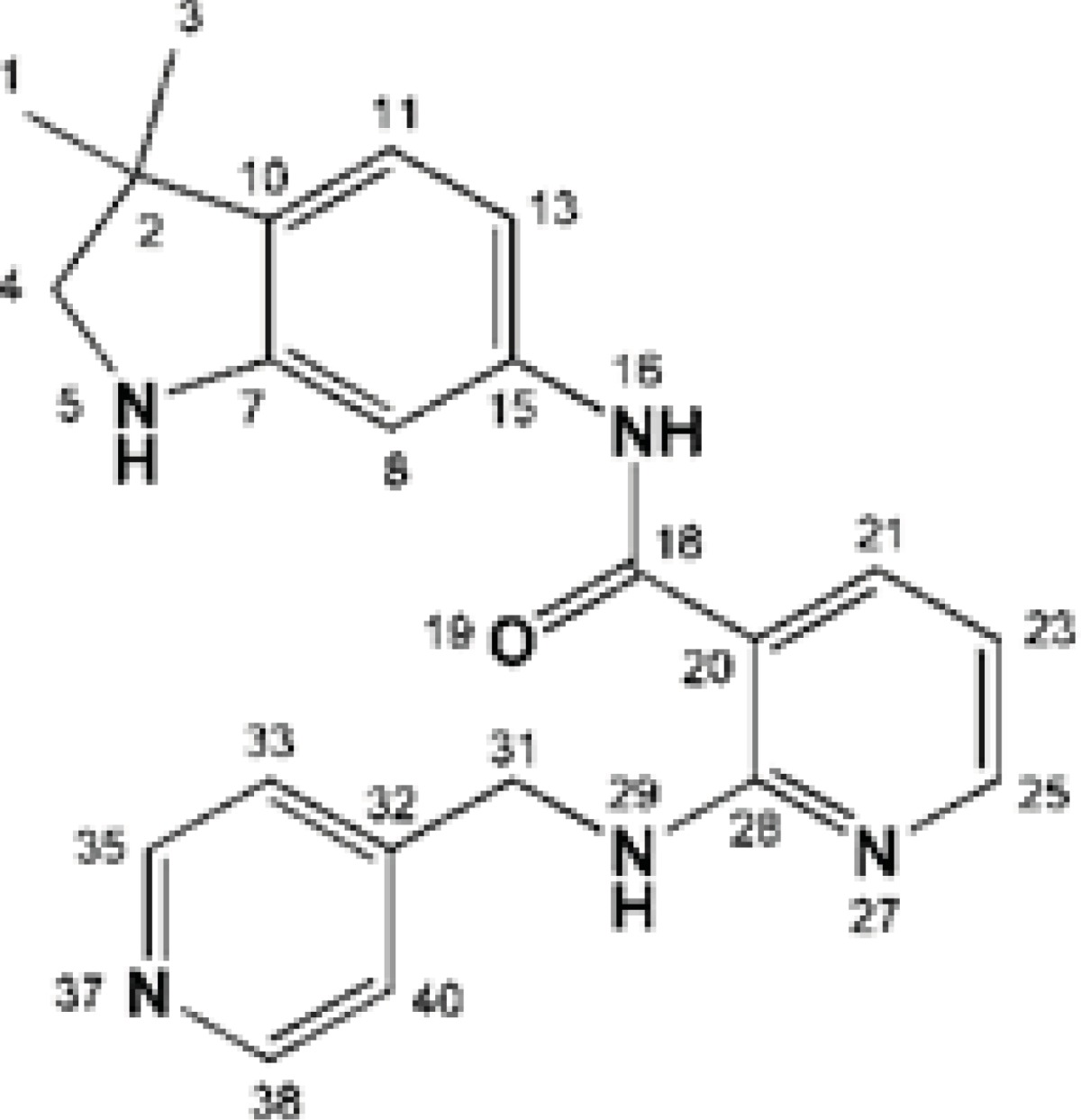

The varied dihedral angles between optimized and crystallographic ligand poses would be expected upon binding to the receptor active site. AMG 709 adapted some torsional distortions to get proper oriented pharmacophoric points. These well-oriented functional groups might be critical in achieving optimum key interactions with the residues of the VEGFR-2 active site. 

Regarding the data in Table 3, some relatively significant angular deviations may be noticed. The observed rotation of C15-C16 bond (Figure 5) let to the noticeable change in C8(13)-C15-N16-C18 dihedral angel (Table 3). This conformational distortion occurred at the amide linker.

All the mentioned conformational changes occurred in the structural moieties participated in interactions with key amino acids of VEFGFR-2 active site (Figure 1).

**Table 3:** Dihedral angles of Motesanib in the optimized and crystallographic (VEGFR-2, PDB code: 3EFL) conformers.

**Dihedral angle**	**Angle (degree)**		**Dihedral angle**	**Angle (degree)**	**Optimized state**
	**Crystallographic state**	**Optimized state**			
H42-C1-C2-C3	-	56.282	C8-C15-N16-C18	-134.086	-178.173
H42-C1-C2-C4	-	-67.538	C13-C15-N16-H17	-	176.423
H42-C1-C2-C10	-	-177.279	C13-C15-N16-C18	52.015	2.511
H43-C1-C2-C3	-	-63.575	C15-N16-C18-O19	1.241	3.4426
H43-C1-C2-C4	-	172.605	C15-N16-C18-C20	-178.813	-177.293
H43-C1-C2-C10	-	62.862	H17-N16-C18-O19	-	-170.398
H44-C1-C2-C3	-	176.567	H17-N16-C18-C20	-	8.8673
H44-C1-C2-C4	-	52.747	N16-C18-C20-C21	15.753	23.472
H44-C1-C2-C10	-	-56.995	N16-C18-C20-C28	-166.812	-158.203
C1-C2-C3-H45	-	178.045	O19-C18-C20-C21	-164.298	-157.260
C1-C2-C3-H46	-	57.839	O19-C18-C20-C28	13.136	21.065
C1-C2-C3-H47	-	-61.358	C18-C20-C21-H22	-	3.080
C4-C2-C3-H45	-	-59.025	C18-C20-C21-C23	177.361	-178.980
C4-C2-C3-H46	-	-179.231	C28-C20-C21-H22	-	-175.305
C4-C2-C3-H47	-	61.572	C28-C20-C21-C23	-0.114	2.635
C10-C2-C3-H45	-	53.925	C18-C20-C28-N27	-177.196	177.765
C10-C2-C3-H46	-	-66.282	C18-C20-C28-N29	2.607	-1.169
C10-C2-C3-H47	-	174.522	C21-C20-C28-N27	0.280	-3.815
C1-C2-C4-N5	-101.759	-88.860	C21-C20-C28-N29	-179.918	177.251
C1-C2-C4-H48	-	31.355	C20-C21-C23-H24	-	-179.516
C1-C2-C4-H49	-	151.806	C20-C21-C23-C25	-0.052	-0.028
C3-C2-C4-N5	133.531	148.232	H22-C21-C23-H24	-	-1.559
C3-C2-C4-H48	-	-91.553	H22-C21-C23-C25	179.979	177.928
C3-C2-C4-H49	-	28.898	C21-C23-C25-H26	-	179.086
C10-C2-C4-N5	15.886	27.029	C21-C23-C25-N27	0.058	-1.787
C10-C2-C4-H48	-	147.243	H24-C23-C25-H26	-	-1.424
C10-C2-C4-H49	-	-92.305	H24-C23-C25-N27	-	177.703
C1-C2-C10-C7	102.711	98.816	C23-C25-N27-C28	0.113	0.682
C1-C2-C10-C11	-74.692	-78.169	H26-C25-N27-C28	-	179.846
C3-C2-C10-C7	-133.134	-136.914	C25-N27-C28-C20	-0.285	2.216
C3-C2-C10-C11	49.463	46.101	C25-N27-C28-N29	179.914	-178.813
C4-C2-C10-C7	-15.307	-17.309	C20-C28-N29-H30	-	-8.165
C4-C2-C10-C11	167.290	165.705	C20-C28-N29-C31	171.865	-177.332
C2-C4-N5-H6	-	-161.533	N27-C28-N29-H30	-	172.857
C2-C4-N5-C7	-12.375	-28.598	N27-C28-N29-C31	-8.334	3.690
H48-C4-N5-H6	-	77.305	C28-N29-C31-C32	94.086	102.346
H48-C4-N5-C7	-	-149.760	C28-N29-C31-H50	-	-135.519
H49-C4-N5-C6	-	-43.223	C28-N29-C31-H51	-	-19.574
H49-C4-N5-C7	-	89.712	H30-N29-C31-C32	-	-66.239
C4-N5-C7-C8	-170.367	-163.627	H30-N29-C31-H50	-	55.896
C4-N5-C7-C10	2.573	18.132	H30-N29-C31-H51	-	171.841
H6-N5-C7-C8	-	-31.016	N29-C31-C32-C33	5.187	-4.398
H6-N5-C7-C10	-	150.744	N29-C31-C32-C40	-173.633	175.611
N5-C7-C8-H9	-	2.343	H50-C31-C32-C33	-	-126.654
N5-C7-C8-C15	171.649	-177.916	H50-C31-C32-C40	-	53.354
C10-C7-C8-H9	-	-179.584	H51-C31-C32-C33	-	116.660
C10-C7-C8-C15	-0.751	0.156	H51-C31-C32-C40	-	-63.331
N5-C7-C10-C2	8.568	0.493	C31-C32-C33-H34	-	0.520
N5-C7-C10-C11	-173.683	177.903	C31-C32-C33-C35	-178.533	-179.807
C8-C7-C10-C2	-177.684	-177.894	C40-C32-C33-H34	-	-179.489
C8-C7-C10-C11	0.066	-0.484	C40-C32-C33-C35	0.297	0.184
C7-C8-C15-C13	1.044	-0.027	C31-C32-C40-C38	178.603	179.705
C7-C8-C15-N16	-172.919	-179.364	C31-C32-C40-H41	-	-0.199
H9-C8-C15-C13	-	179.715	C33-C32-C40-C38	-0.223	-0.288
H9-C8-C15-N16	-	0.378	C33-C32-C40-H41	-	179.809
C2-C10-C11-H12	-	-3.188	C32-C33-C35-H36	-	-179.948
C2-C10-C11-C13	177.471	177.413	C32-C33-C35-N37	-0.201	0.009
C7-C10-C11-H12	-	-179.917	H34-C33-C35-H36	-	-0.274
C7-C10-C11-C13	0.316	0.684	H34-C33-C35-N37	-	179.683
C10-C11-C13-H14	-	179.731	C33-C35-N37-C38	0.036	-0.095
C10-C11-C13-C15	-0.019	-0.558	H36-C35-N37-C38	-	179.863
H12-C11-C13-H14	-	0.323	C35-N37-C38-H39	-	-179.957
H12-C11-C13-C15	-	-179.966	C35-N37-C38-C40	0.031	-0.018
C11-C13-C15-C8	-0.654	0.224	N37-C38-C40-C32	0.070	0.214
C11-C13-C15-N16	173.284	179.508	N37-C38-C40-H41	-	-179.882
H14-C13-C15-C8	-	179.938	H39-C38-C40-C32	-	-179.849
H14-C13-C15-N16	-	-0.777	H39-C38-C40-H41	-	0.055
C8-C15-N16-H17	-	-4.261			

## Conclusion

Amino acid decomposition analysis provided further insight into the effect of individual amino acid residues on Motesanib/VEGFR-2 binding profile. Such structure-based studies may serve as efficient analyzing tools in evaluating the pharmacophore models. Owing to the prominent role of electrostatic forces in initial ligand-receptor interactions, charge-assisted H-bonds and cation-pi interactions need to be particularly attended. In the case of Cys919, the estimated binding energy could further confirm the role of this key amino acid in contribution to H-bond interactions reported for various VEGFR inhibitors. We could further demonstrate that Motesanib does not necessarily bind to the receptor in its optimum conformation state.
